# DDX5 potentiates HIV-1 transcription as a co-factor of Tat

**DOI:** 10.1186/s12977-020-00514-4

**Published:** 2020-03-30

**Authors:** Nyaradzai Sithole, Claire A. Williams, Truus E. M. Abbink, Andrew M. L. Lever

**Affiliations:** 1 Department of Medicine, University of Cambridge, Addenbrooke’s Hospital, Cambridge, CB2 0QQ UK; 2 Department of Medicine, National University of Singapore, Singapore, 119228 Singapore; 3 Present Address: Department of Microbiology, Specialist Virology Centre, Norfolk and Norwich University Hospitals, Norwich, UK; 4 Present Address: Department of Paediatrics, Child Neurology, Centre for Childhood White Matter Disorders, VU University Medical Centre, Amsterdam, The Netherlands

**Keywords:** HIV-1, Tat, Transcription, P-TEFb, DEAD Box helicases, DDX5, siRNA, HEXIM1/2

## Abstract

**Background:**

HIV-1 does not encode a helicase and hijacks those of the cell for efficient replication. We and others previously showed that the DEAD box helicase, DDX5, is an essential HIV dependency factor. DDX5 was recently shown to be associated with the 7SK snRNP. Cellular positive transcription elongation factor b (P-TEFb) is bound in an inactive form with HEXIM1/2 on 7SK snRNP. The Tat/P-TEFb complex is essential for efficient processivity of Pol II in HIV-1 transcription elongation and Tat competes with HEXIM1/2 for P-TEFb. We investigated the precise role of DDX5 in HIV replication using siRNA mediated knockdown and rescue with DDX5 mutants which prevent protein–protein interactions and RNA and ATP binding.

**Results:**

We demonstrate a critical role for DDX5 in the Tat/HEXIM1 interaction. DDX5 acts to potentiate Tat activity and can bind both Tat and HEXIM1 suggesting it may facilitate the dissociation of HEXIM1/2 from the 7SK-snRNP complex, enhancing Tat/P-TEFb availability. We show knockdown of DDX5 in a T cell line significantly reduces HIV-1 infectivity and viral protein production. This activity is unique to DDX5 and cannot be substituted by its close paralog DDX17. Overexpression of DDX5 stimulates the Tat/LTR promoter but suppresses other cellular and viral promoters. Individual mutations of conserved ATP binding, RNA binding, helicase related or protein binding motifs within DDX5 show that the N terminal RNA binding motifs, the Walker B and the glycine doublet motifs are essential for this function. The Walker A and RNA binding motifs situated on the transactivation domain are however dispensable.

**Conclusion:**

DDX5 is an essential cellular factor for efficient HIV transcription elongation. It interacts with Tat and may potentiate the availability of P-TEFb through sequestering HEXIM1.

## Background

DEAD box RNA helicases are ubiquitous cellular proteins with protean functions. They derive their name from the first letter amino acid sequence of their conserved Walker B motif. They bind nucleic acids and are involved in many cellular processes including transcription, pre-mRNA splicing, cellular differentiation, translation, RNA/protein stabilization and remodelling [1, 2].

Unlike a number of viruses that encode their own RNA helicases (such as the NS3 protein of Hepatitis C) [3, 4] Human immunodeficiency virus type 1 (HIV-1) does not, instead utilizing cellular helicases as essential cofactors at several stages of its replication cycle. The first of these to be identified was DDX3 which facilitates Rev mediated export of unspliced and partially spliced viral transcripts [5]. Subsequently DDX1 was shown to be a cofactor of Rev [6] while RNA helicase A (RHA) promotes HIV-1 reverse transcriptase, transcription and translation [6–10].

We previously published a comprehensive siRNA knockdown screen of a library of 59 human cellular helicases. Amongst those identified as important HIV dependency factors were the two closely related DEAD box helicases, DDX5 and DDX17 [11]. Using siRNA specific for knockdown of DDX5 and rescue with an siDDX5 resistant construct we have confirmed this phenotype in the physiologically relevant Jurkat cell line; knockdown having no detectable adverse effect on cellular viability.

DDX5 and DDX17 share 90% homology in their core regions and 60% and 30% similarity in their N and C termini respectively [12]. Because of their similarities and close interplay, they are sometimes considered as functional homologs although each has been shown to have certain distinct roles [13–15]. The two proteins can exist as monomers, homodimers or heterodimers [16]. The complexity of their interaction is compounded by an as yet unexplained mechanism whereby overexpression of DDX5 results in a reduction in DDX17 [17]. We showed that DDX17 is a critical splicing factor for HIV-1 controlling the A4/5 splice acceptor site and that this function is independent of DDX5 [18].

Previous studies on DDX5 in the context of HIV-1, have produced conflicting results. siRNA mediated knockdown was shown to increase HIV CA-p24 production and infectivity, likely due to the documented increase in DDX17 expression noted in DDX5 depleted cells [19]. However in a different study in which DDX5 was identified as a co-factor of Rev, knockdown apparently reduced HIV-1 CA-p24 production and viral infectivity [20]. Given these findings and the evidence we had suggesting that DDX5 is an HIV-1 dependency factor we sought to clarify the role of DDX5 in HIV-1 replication and whether its actions were dependent or independent of DDX17. Our evidence suggests that, despite their documented interplay in the cell, DDX5 and DDX17 have quite distinct and independent effects on HIV.

The integrated HIV-1 provirus is transcribed by cellular RNA polymerase II (Pol II) and the mechanism of transcriptional initiation is similar to cellular genes. It differs in that the switch from initiation to elongation at the HIV-1 promoter is dependent on the viral transactivator Tat, which recruits P-TEFb to the stalled Pol II [21]. P-TEFb is composed of cyclinT1/2 (CycT1/2) and cyclin-dependent kinase 9 (Cdk9) [22–24]. The kinase catalytic domain phosphorylates the Ser2 residues in the C-terminal domain (CTD) of Pol II [24–26]. Formation of the Tat/P-TEFb complex is critical for efficient HIV-1 transcription elongation [23]. In its absence Pol II terminates prematurely soon after the TAR region due to poor processivity of the RNA Pol II/transcription complex [23, 27, 28].

The majority of P-TEFb is sequestered, bound to HEXIM1 in the transcriptionally inactive 7SK snRNP complex, composed of 7SK snRNA, hexamethylene bisacetamide (HMBA) induced protein 1 (HEXIM1), methylphosphate capping enzyme (MEPCE) and Larp7/Pip7S, in addition to P-TEFb [24, 26, 29–31]. Dynamic remodelling of 7SK snRNP regulates the availability of active P-TEFb [32]. A recent study has shown that KAP1 recruits the 7SK snRNP complex to promoter proximal regions and, using mass spectrometry, interactors of LARP7 were identified, amongst which was DDX5 [33].

Tat competes with HEXIM1 to promote the release of P-TEFb increasing the available pool of active P-TEFb [30, 34]. The Tat binding region on 7SK snRNA is embedded within the HEXIM1 binding domain and is structurally and functionally indistinguishable from the Tat/TAR binding region [35]. Tat forms two biochemically distinct complexes, Tatcom1 and Tatcom2. Formation of Tatcom2 which is devoid of HEXIM1 is thought to be either by Tat displacing HEXIM1 from 7SK snRNP by direct competition for 7SK RNA binding or by Tat being recruited to 7SK RNA during formation of 7SK snRNP [34]. Although it is not known how Tat eventually dissociates from 7SK snRNA to enable it to bind P-TEFb, it is envisaged that Tat interacts dynamically with cellular factors that facilitate not only its binding with 7SK snRNA but also its release from the 7SK snRNA and subsequent presentation to the P-TEFb complex.

Here, we show that HIV-1 Tat can interact with DDX5 and that, in the presence of DDX5, Tat has a competitive advantage over HEXIM1 for recruitment of P-TEFb. We show that DDX5 interacts with HEXIM1. This could potentially sequester it as a mechanism of potentiating HIV-1 transcription elongation. Using mutational analysis of individual motifs in the context of rescue experiments, the domains of DDX5 essential for its role in HIV-1 replication have been identified. We also demonstrate that DDX5 acts independently of DDX17.

## Results

### DDX5 is essential for HIV-1 CA-p24 production and infectivity

**Fig. 1:**
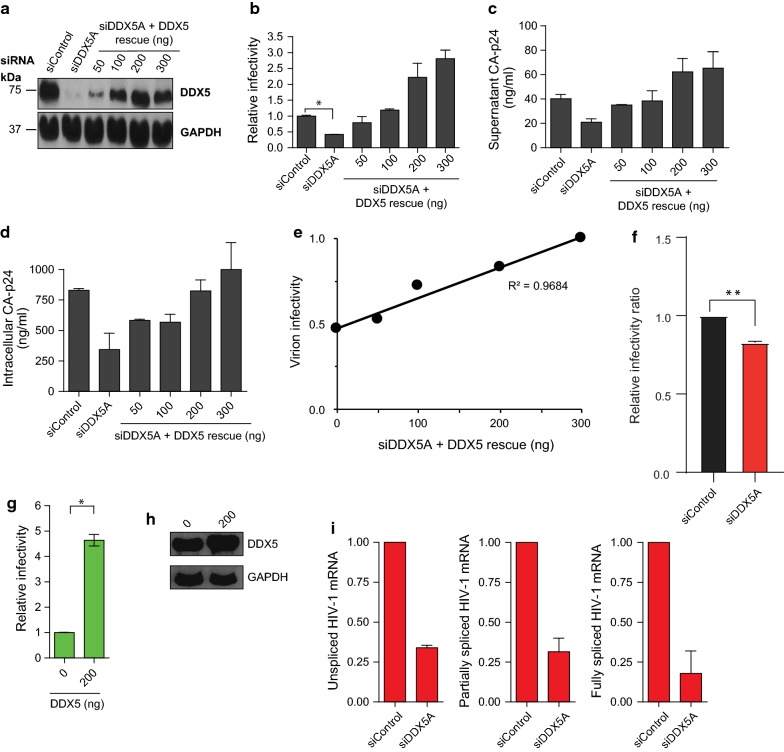
Effect of DDX5 knockdown and rescue on HIV-1 infectivity. **a** Western blot analysis of DDX5 knockdown and rescue. HIV-1 proviral clone pLAI, and siDDX5A with or without increasing concentrations of siDDX5A rescue expressor were transfected into HeLa cells. **b** HIV-1 infectivity following DDX5 knockdown and rescue. Cells were transfected with siDDX5A and then a second round of siRNA together with pLAI, with or without increasing concentrations of siDDX5A rescue construct. Supernatant was used to infect TZM-bl cells. Relative infectivity was calculated by dividing individual raw luminescence data with that from siControl treated cells. **c** Supernatant from **b** was harvested and CA-p24 quantified by ELISA. **d** Cell lysates from **b** were harvested and CA-p24 quantified by ELISA. Each graph is a representative of three independent experiments done in triplicate. **e** Linear regression of mean virion infectivity from triplicate samples on dose of DDX5A rescue plasmid are shown. **f** Per virion infectiousness was calculated using the virion infectivity (infectivity divided by supernatant CA-p24) and normalised to siControl treated cells. **g** Supernatant was harvested 48 h after co-transfection of a constant amount of pLAI with increasing concentrations of Myc-DDX5 and used to infect TZM-bl cells. The graph shown is representative of at least two independent experiments done in duplicate. Data is represented as mean of duplicate samples ± SEM. **h** Cell lysates from **g** were harvested and subjected to western blotting. **i** RT-qPCR analysis of unspliced, partially and fully spliced HIV-1 mRNA from left to right respectively. Cells were sequentially transfected with siControl or siDDX5A and then a second round of siRNA together with pLAI. Data shown is representative of three independent repeats, with triplicate samples for each siRNA. Data has been normalised to siControl. Error bars represent ± SEM. Values are scored as a fold-change relative to that of siControl treated cells. Statistical significance: *p < 0.05. See also Additional file 1: Figure S1

We used siDDX5A to knockdown, and siDDX5A resistant expression constructs to rescue, expression of DDX5 in HeLa cells. Cells were sequentially transfected with siDDX5A followed by a second round of siRNA together with the replication competent HIV-1 proviral clone pLAI, with or without increasing concentrations of an siDDX5A rescue plasmid. Knockdown and rescue of DDX5 were confirmed by western blotting (Fig. 1a). There was no detectable cellular toxicity following treatment with siDDX5A with or without increasing concentrations of rescue plasmid (Additional file 1: Fig. S1a).

Supernatants from these transfected cells were used to infect the CD4+TZM-bl indicator cell line. DDX5 knockdown reduced HIV-1 infectivity and both intracellular and supernatant CA-p24 levels (Fig. 1b–d). All three parameters were successfully restored upon co-expression of siDDX5A with the siDDX5A resistant DDX5 expressor, confirming that the observed phenotypes were specifically due to DDX5 depletion.

Expression of the rescue plasmid not only restored viral infectivity, intracellular and supernatant CA-p24 but could augment it above the level seen in siControl treated cells (Fig. 1b–d). We wondered whether this was due to a quantitative increase in virion production or if the virions produced had a higher specific infectivity. The increased infection seen on rescue may have been unique to some characteristic of expression from the rescue plasmid or intrinsic to wild type DDX5. To address this, we performed a DDX5 overexpression experiment. HeLa cells were co-transfected with increasing amounts of DDX5 plasmid and a constant amount of pLAI. Virion infectivity was determined by dividing infectious virus release (determined using TZM-bl cells) by supernatant CA-p24 (quantified by ELISA) and expressed as a fraction of maximum. Linear regression of mean virion infectivity from triplicate samples on dose of DDX5A rescue plasmid are shown in Fig. 1e. Relative virion infectivity was measured by dividing the raw infectivity data for each condition with the infectivity data from siControl treated cells. The relative virion infectivity revealed that siDDX5 treated cells have significant reduction in the infectiousness of virions produced (Fig. 1f). Overexpression of DDX5 resulted in a significant (fivefold) increase in HIV-1 infectivity (Fig. 1g, h). We postulate that the apparent increased infectivity per virion might be due to a number of causes including increased HIV transcription or more efficient viral RNA modulation or stability. Knockdown of DDX5 resulted in a 3-fivefold reduction of each of the different HIV-1 mRNA species (Fig. 1i) consistent with global inhibition of viral RNA production rather than differential effects on individual spliced forms.

### Importance of DDX5 for HIV-1 replication in a T cell line

**Fig. 2:**
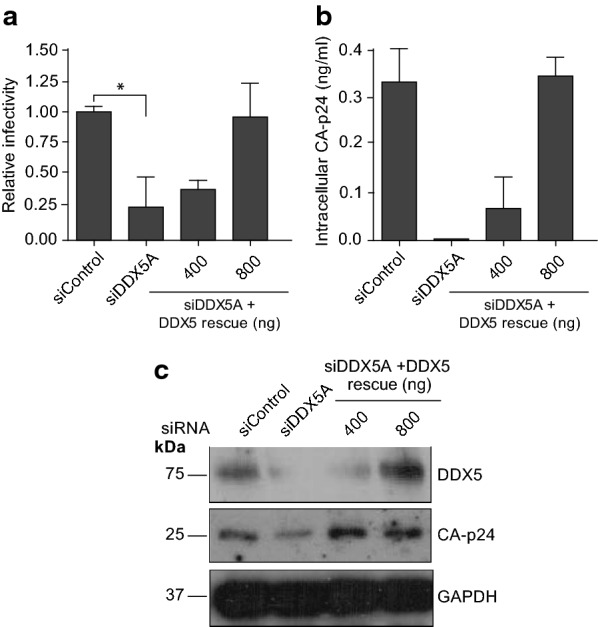
DDX5 is essential for HIV-1 infectivity and CA-p24 production in Jurkat cells. **a** HIV-1 infectivity following DDX5 knockdown and rescue in Jurkat cells. Supernatant was harvested 96 h post nucleofection and used to infect TZM-bl cells. **b** Cell lysates were harvested 96 h post nucleofection and CA-p24 quantified by ELISA. Bars represent mean of duplicate samples ± SEM. **c** Western blot showing the expression of DDX5 following knockdown and rescue

To ensure that our findings were relevant to cell lines infectable by HIV-1, we carried out nucleofection of Jurkat cells using the HIV proviral clone pLAI together with siDDX5A with or without increasing concentrations of the siDDX5A rescue plasmid. The same trend that we observed in HeLa cells was recapitulated. siRNA knockdown of DDX5 in Jurkat cells reduces HIV-1 infectivity and CA-p24 production; both characteristics are rescued upon expression of the rescue plasmid (Fig. 2a, b). The siDDX5A rescue plasmid was well expressed in Jurkat cells (Fig. 2c).

### DDX5 interacts with both Tat and HEXIM1 and facilitates efficient HIV-1 transcription

DEAD box helicases typically influence either transcriptional or post-transcriptional events [36] and there is published evidence that DDX5 can affect transcription of cellular genes [37, 38].

**Fig. 3:**
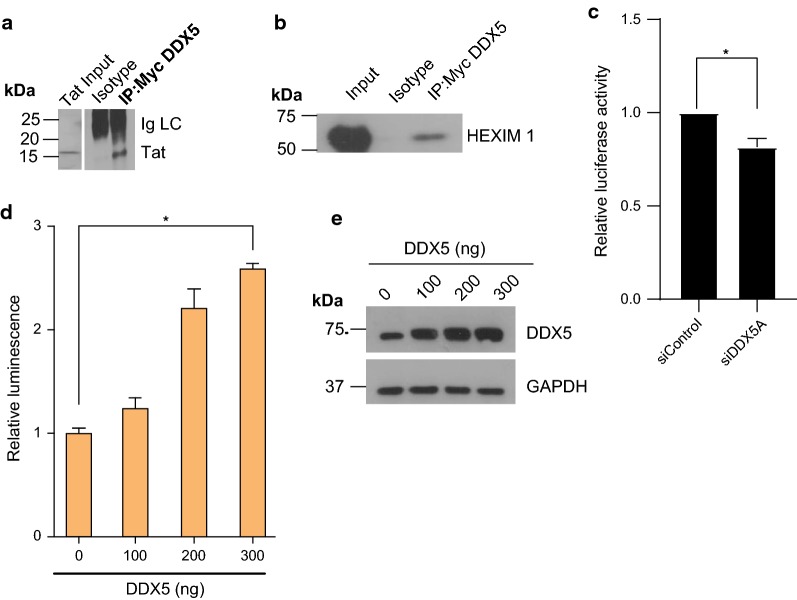
DDX5 is specifically required for HIV-1 transcription and interacts with HIV Tat and cellular HEXIM1. **a** Myc-DDX5 was co-transfected with HIV-1 replication competent proviral construct (pLAI). Cells were harvested after 48 h and subjected to co-immunoprecipitation followed by western blot for HIV Tat. DDX5 co-immunoprecipitates with Tat. Hela whole cell lysates from cells transfected with replication competent HIV-1 proviral plasmid, were run in parallel as positive control for HIV Tat, to the immunoprecipitation treated samples. The blot is a representative of three independent experiments. **b** Myc-DDX5 was co-transfected with HIV-1 proviral construct (pLAI). Cells were harvested after 48 h and subjected to co-immunoprecipitation followed by western blot for HEXIM1. Representative western blot of three independent experiments showing that DDX5 co-immunoprecipitates with HEXIM1. **c** TZM-bl cells were transfected with constant amount of Tat expressor plus either siControl or siDDX5A. Cell lysates were harvested after 72 h and luciferase activity measured using the luciferase assay. The graph is a representative of three independent experiments done in triplicate. Bars represent mean of triplicate samples ± SEM. **d** DDX5 potentiates Tat dependent, HIV-1 LTR-driven, firefly luciferase activity. TZM-bl cells were co-transfected with pcDNA3.0-Tat and increasing concentration of Myc-DDX5. Cells were harvested after 48 h and luciferase activity measured. The graph shown is a representative of three independent experiments done in duplicate ± SEM. **e** Western blot showing the expression levels of DDX5 in TZM-bl cells. Statistical significance: *P < 0.05. See also Additional file 2: Figure S2 and Additional file 3: Figure S3

Previous in vitro studies have revealed that Tat can interact with DDX5 [39]. However, to our knowledge there are no studies showing this in a cellular context. To seek evidence of interaction between DDX5 and Tat or the P-TEFb complex we performed co-immunoprecipitations. Myc tagged DDX5 was co-transfected with HIV-1 replication competent proviral plasmid in HeLa cells. Due to the fact that there is little *de*-*novo* synthesis of Tat we used transfected whole cell lysates as positive control. HIV Tat co-immunoprecipitates with DDX5 (Fig. 3a), but not the P-TEFb components Cyclin T1 and CDK9 (Additional file 2: Fig. S2a, b). Given that HEXIM1 competes with Tat for P-TEFb at the 7SK snRNP, we wondered if DDX5 could interact with HEXIM1. We found that DDX5 co-immunoprecipitates with HEXIM1 (Fig. 3b). To seek any potentiating effect of DDX5 on Tat, we transfected siDDX5 together with a Tat expressor in TZM-bl indicator cells. Knockdown of DDX5 significantly reduced the Tat driven luciferase activity (Fig. 3c). To further establish that DDX5 potentiates Tat, we performed a DDX5 dose dependence experiment by co-transfecting a constant concentration of Tat expressor with increasing concentrations of DDX5 in TZM-bl cells. Overexpression of DDX5 resulted in a significant increase in the Tat driven luciferase activity (Fig. 3d, e). Further experiments to confirm that DDX5 potentiates Tat driven LTR activity were carried out by co-transfection of increasing concentration of DDX5 with constant amount of Tat expressor and LTR- driven luciferase reporter construct in Hela cells (Additional file 3: Fig. S3a, b). Overexpression of DDX5 significantly increased Tat driven luciferase activity and knockdown of DDX5 reduced Tat driven luciferase activity (Additional file 3: Fig. S3a, c).

DDX5 thus can interact with Tat and HEXIM1 and enhances Tat dependent transcription. To determine whether DDX5 potentiation of Tat is HIV-1 LTR promoter specific and not due to non-specific transcriptional enhancing effects of DDX5, we transfected in independent experiments, three different luciferase reporter constructs under different promoters. Overexpression of DDX5 assessed by Western blot (Additional file 3: Fig. S3g, h and i) has a significant dominant negative effect on human elongation factor driven luciferase activity (Additional file 3: Fig. S3d), CMV driven luciferase activity (Additional file 3: Fig. S3e) and T7 promoter driven luciferase activity (Additional file 3: Fig. S3f).

### The role of DDX5 in HIV-1 is independent of its paralog DDX17

**Fig. 4:**
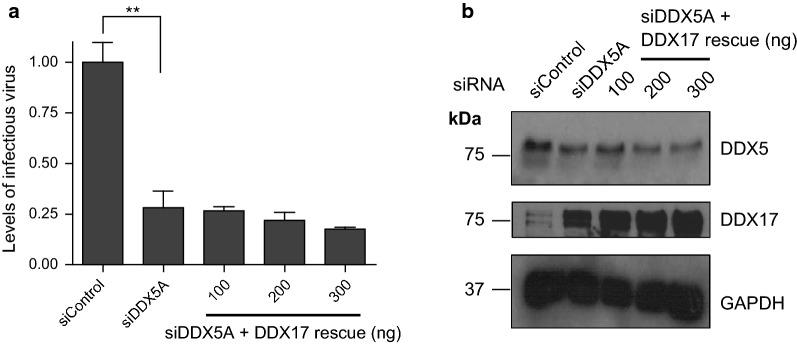
DDX5 acts independently of DDX17. **a** Cells were sequentially transfected with siDDX5A and then a second round of siRNA together with pLAI, with or without increasing concentrations of a DDX17 expressor. Supernatant was harvested after 48 h and used to infect TZM-bl cells. The graph shown is a representative of three independent experiments done in triplicate. Bars represent mean of triplicate samples ± SEM. **b** Knockdown and rescue was determined by western blotting for DDX5 and DDX17 expression. Statistical significance: **P < 0.01. See also Figure S4

Given the intimate relationship between DDX5 and DDX17 we next queried whether the transcriptional enhancement of DDX5 on HIV-1 was dependent or independent of its paralog. We transfected either Myc tagged DDX17 or a Myc tagged DDX5 expressor in HeLa cells. Cells were harvested, and nuclear extracts prepared followed by either pulldown of Myc-DDX17 or Myc-DDX5 and immunoblotting for DDX5 or DDX17 respectively. We could confirm by co-immunoprecipitation the published findings that DDX5 interacts with DDX17 (Additional file 4: Fig. S4a, b). Myc-DDX17 or Myc-DDX5 was transfected in HeLa cells and cell lysates harvested after 48 h for co-immunoprecipitation. Additional file 4: Figure S4a shows pulldown of Myc-DDX17 followed by immunoblotting for DDX5. Additional file 4: Figure S4b shows pulldown of Myc-DDX5 followed by immunoblotting for DDX17. siRNA knockdown of DDX5 up-regulates levels of DDX17 although this is non reciprocal [19, 40]. Overexpression of DDX5 downregulates endogenous DDX17 (Additional file 4: Fig. S4c) [17]. Our findings that overexpression of DDX5 reduces DDX17 expression yet leads to an increase in HIV-1 replication supports the case that DDX5 has a direct role in HIV-1 independent of DDX17. To further distinguish the roles of these two proteins, we attempted to rescue DDX5 knockdown by overexpression of DDX17 however this failed to restore HIV-1 infectivity (Fig. 4a, b). These findings underscore that DDX5 is a critical factor for HIV-1 replication independent of DDX17.

### DDX5 utilises distinct RNA binding motifs to facilitate efficient HIV-1 replication

We investigated whether the transcriptional activity of DDX5 in HIV-1 replication was dependent on its nucleic acid binding or modifying functions. We introduced individual point mutations in known RNA and ATPase binding motifs and cloned each mutant into the siDDX5A rescue backbone. We tested individual mutant constructs in the context of HIV-1 replication by measuring their ability to restore viral infectivity and CA-p24 production in DDX5 depleted cells.

**Fig. 5:**
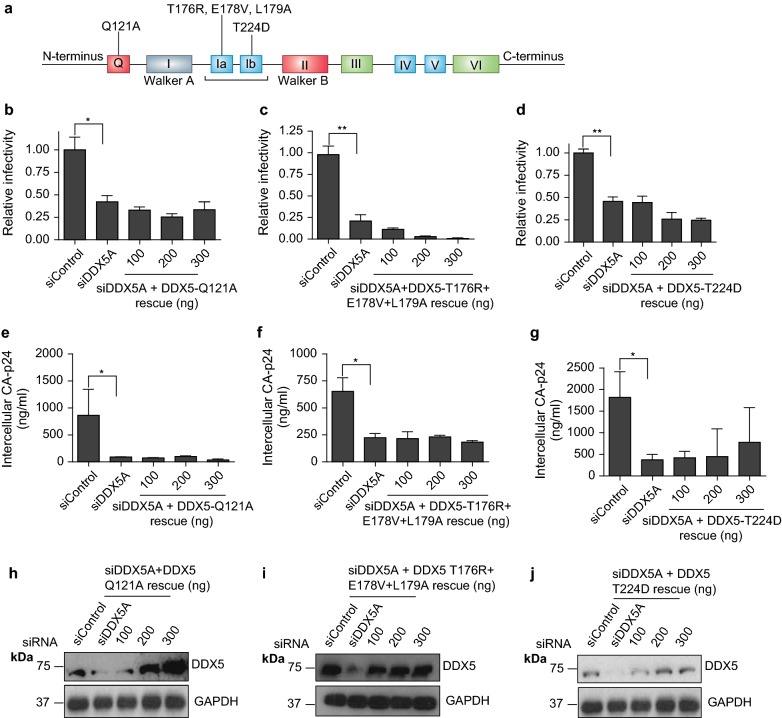
DDX5 N terminal RNA binding motifs are indispensable in HIV-1 replication. **a** Schematic representation of DDX5 and the annotated individual point mutations that are defective for ATPase and RNA binding activities in the Q motif and motifs 1a and 1b. **b** HIV-1 infectivity following DDX5 depletion and rescue with siDDX5A-resistant, Q motif mutated plasmid DDX5-Q121A. Supernatant from transfected cells was used to infect TZM-bl cells. **c** HIV-1 infectivity following DDX5 depletion and rescue with siDDX5A-resistant, motif 1a mutated plasmid, DDX5-T176R + E178V + L179A. Supernatant from transfected cells was used to infect TZM-bl cells. **d** HIV-1 infectivity following DDX5 depletion and rescue with siDDX5A-resistant, motif mutated plasmid, DDX5-T224D. Supernatant from cells transfected with siDDX5A and then a second round of siRNA together with pLAI, with or without increasing concentrations of DDX5-T224D construct, was used to infect TZM-bl cells. **e** Cell lysates from **b** were harvested and CA-p24 quantified by ELISA. **f** Cell lysates from **c** were harvested and CA-p24 quantified by ELISA. **g** Cell lysates from **d** were harvested and CA-p24 quantified by ELISA. Each graph is a representative of at least two independent experiments done in triplicate. Bars represent mean of triplicate samples ± SEM. **h** Western blot showing the expression of DDX5 after knockdown and rescue with DDX5-Q121A. **i** Western blot showing the expression of DDX5 after knockdown and rescue with DDX5-T176R + E178V + L179A. **j** Western blot showing the expression of DDX5 after knockdown and rescue with DDX5-T224D. Statistical significance *P < 0.05, **P < 0.01. See also Additional file 4: Figure S4

The Q motif is necessary for binding of ssRNA and, through its interaction with motif 1a, it is important for ATP binding, acting as a regulator of ATPase activity. Substituting the highly conserved glutamine residue (aa121) by alanine abrogates ATP/RNA binding [41, 42]. The siDDX5A resistant DDX5-Q121A construct failed to restore production of infectious virus (Fig. 5a, b).

Motif 1a (PTRELA) and 1b (TPGR) are necessary for RNA binding in conjunction with motifs IV and V [43]. Introducing individual point mutations within either motif 1a (PTRELA to PRRVAA) or motif 1b (TPGR to DPGR) abrogates ATP/helicase and RNA binding activities respectively [44]. Furthermore, the mutant TPGR to DPGR has a detrimental effect on ATPase, RNA crosslinking and helicase activities [44]. In the context of rescue experiments, both mutants fail to restore HIV-1 infectivity (Fig. 5c, d). All three mutants, DDX5-Q121A, DDX5-T176R+E178V+L179A and DDX5-T224D failed to restore CA-p24 production, despite adequate expression (Fig. 5e–j).

Thus, individually abrogating the Q motif or motifs 1a and 1b in the rescue construct failed to restore viral infectivity supporting the notion that they are critical components of DDX5 function in the HIV replication cycle.

Surprisingly expression of the RNA binding defective mutants DDX5-S279L and DDX5-R431Q, situated within the transactivation domain of DDX5, restored HIV-1 infectivity (Additional file 5: Fig. S5a–e). Thus, unlike those at the N terminus, the RNA binding motifs within the transactivation domain are not important for HIV transactivation. Attempts to truncate the transactivation domain and use the resulting mutant in RNAi rescue experiments were not successful as the mutant could not be expressed at comparable levels to the wild type.

### DDX5 DEAD box signature motif (Walker B) is essential for HIV-1 replication

**Fig. 6:**
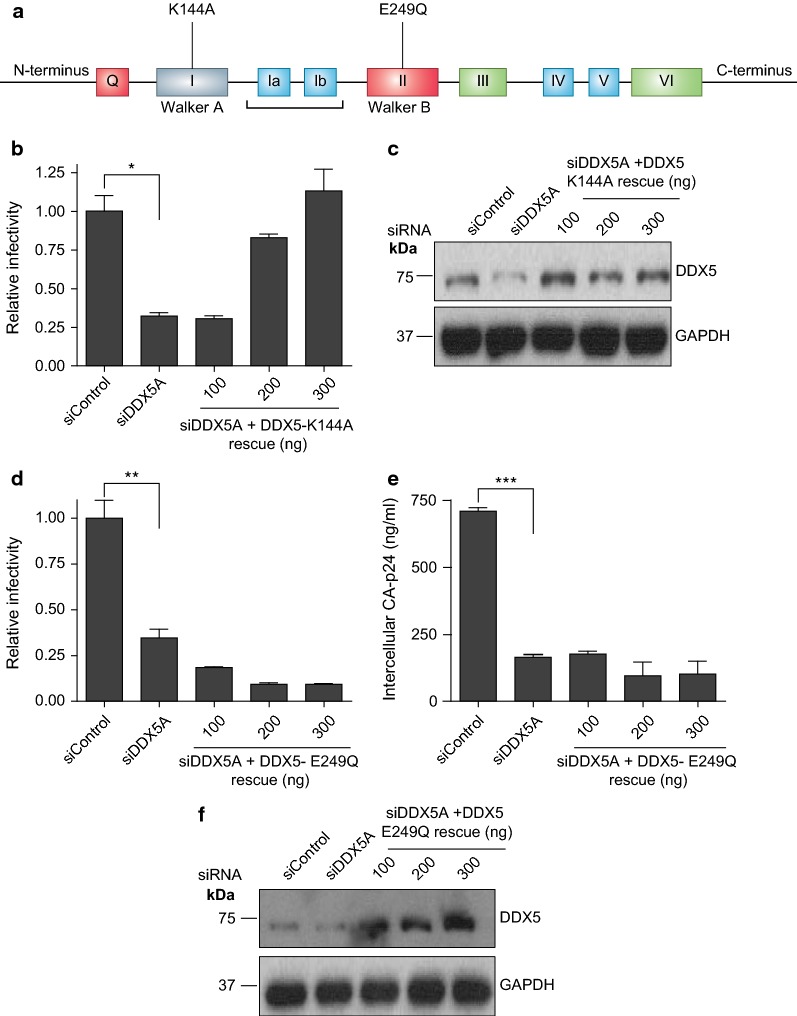
DDX5 Walker A is dispensable but Walker B (DEAD) helicase activity is required. **a** Schematic representation of DDX5 and the annotated individual point mutations in the Walker A and Walker B (DEAD) motifs that are defective for ATPase and helicase activity respectively. **b** HIV-1 infectivity following DDX5 depletion and rescue with siDDX5A-resistant, Walker A motif mutated plasmid, DDX5-K144A. Supernatant from transfected cells was used to infect TZM-bl cells. **c** Western blot showing the expression of DDX5 after knockdown and rescue with DDX5-K144A. **d** HIV-1 infectivity following DDX5 depletion and rescue with siDDX5A-resistant, Walker B motif mutated plasmid, DDX5-E249Q. Supernatant from transfected cells was used to infect TZM-bl cells. **e** Cell lysates from **d** were harvested and CA-p24 quantified by ELISA. Each graph is a representative of at least two independent experiments done in triplicate. Bars represent mean of triplicate samples ± SEM. Statistical significance *P < 0.05, **P < 0.01, ***P < 0.0001. **f** Western blot showing the expression of DDX5 after knockdown and rescue with DDX5-E249Q

We introduced siRNA-resistant DDX5 constructs with either abrogated Walker A or Walker B activity to DDX5 depleted cells (Fig. 6a). Mutating the conserved lysine to alanine residue in Walker A abolishes its ATPase activity by reducing the affinity for and the rate of hydrolysis of ATP [45]. However, the Walker A motif which is crucial in ATPase and helicase activities is dispensable for the role of DDX5 in HIV-1 replication since a siDDX5A rescue mutant with abrogated Walker A (DDX5-K144A) activity successfully restores viral infectivity (Fig. 6b). Expression levels of the mutant did not vary significantly (Fig. 6c).

The Walker B motif (DEAD) is well characterised for its role in helicase activity [1]. A point mutation (DEAD to DQAD) that abolishes helicase activity (siDDX5A resistant DDX5-E249Q) (Fig. 6a), failed to restore viral infectivity and CA-p24 production despite adequate expression (Fig. 6d–f), indicating the helicase activity of the Walker B motif is essential for HIV-1 replication.

### Disruption of the DDX5 protein/protein interaction domain inhibits HIV-1 replication

**Fig. 7:**
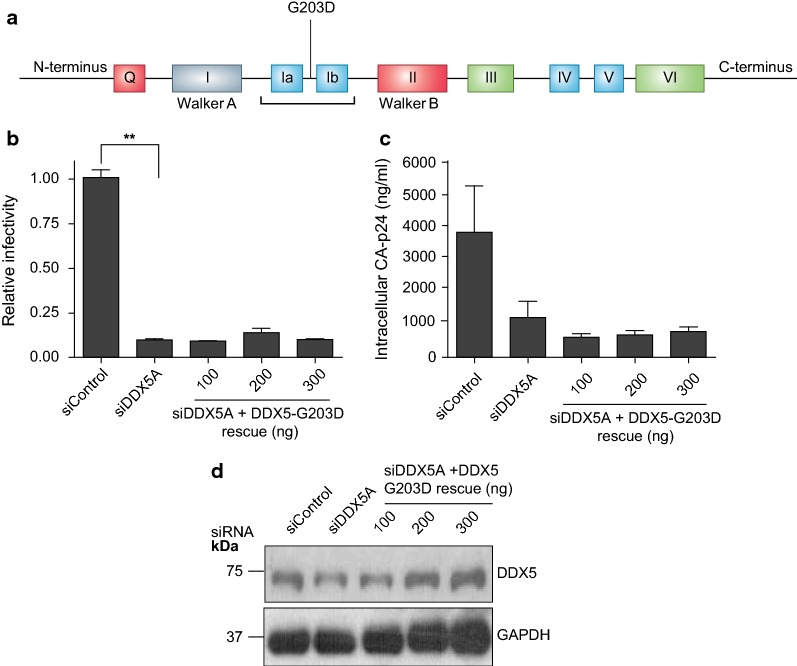
DDX5 glycine doublet is essential for efficient HIV-1 replication. **a** Schematic representation of DDX5 and the annotated individual point mutations in the glycine doublet motif that is defective for ATPase/helicase activities and protein–protein interactions. **b** HIV-1 infectivity following DDX5 depletion and rescue with siDDX5A-resistant, glycine doublet motif mutated plasmid, DDX5-G203D. Supernatant from transfected cells was used to infect TZM-bl cells. **c** Cell lysates from **b** were harvested and CA-p24 quantified by ELISA. Each graph is a representative of at least two independent experiments done in triplicate. Bars represent mean of triplicate samples ± SEM. Statistical significance **P < 0.01. **d** Western blot showing the expression of DDX5 after knockdown and rescue with DDX5-G203D

DEAD box helicases share a highly conserved glycine doublet, thought to facilitate the formation of a sharp turn within the loop between motif 1a and motif 1b. It is essential for protein–protein interactions with other proteins [1] and vital for the latter motifs to retain RNA binding activity. Substituting the second glycine residue with aspartic acid disrupts the formation of the turn. This mutant has also been shown to lack ATPase and RNA helicase activity but retains ATP binding activity [44]. We generated DDX5-G203D (Fig. 7a) to interrogate the glycine doublet. Despite DDX5-G203D being well expressed, following depletion of endogenous DDX5, it failed to restore viral infectivity and CA-p24 production (Fig. 7b–d).

The glycine doublet is critical for DDX5 to facilitate its role in HIV-1 transcription. However, our experimental design does not differentiate whether failure of DDX5-G203D mutant to restore viral infectivity and CA-p24 production is a result of disruption of its protein–protein interaction activity or RNA binding.

### DDX5 interaction with HEXIM1 is mediated via RNA binding and Walker B motif

**Fig. 8:**
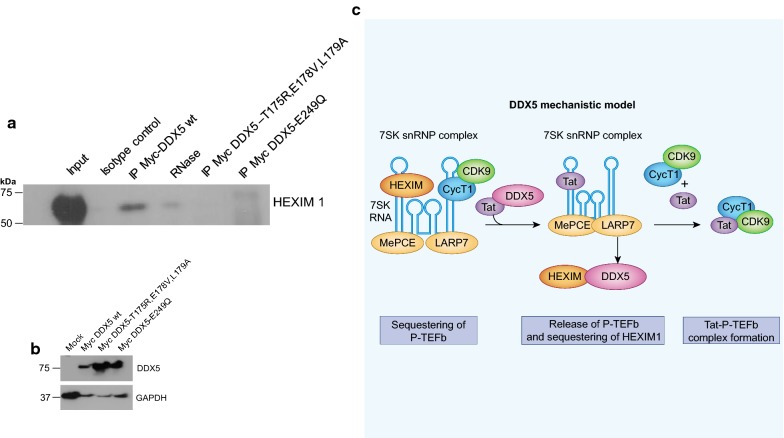
DDX5 RNA binding motif 1a and the Walker B motif are essential for interaction with HEXIM1. **a** HeLa cells were co-transfected with different Myc-tagged DDX5 expressors (DDX5 wild type, DDX5-T176R + E178V + L179A and DDX5-E249Q). Cell lysates were harvested 48 h post co-transfection for co-immunoprecipitation. A Myc specific antibody or isotype matched control antibody was used for pull-down. Samples were subjected to SDS-PAGE and western blotting by probing for HEXIM1. To further validate that the interaction of DDX5 with HEXIM1 is RNA dependent, samples from Myc-DDX5 wild type were treated with RNase enzyme. The western blot shown is a representative from three independent experiments. **b** Western blot from whole cell lysates transfected with the different Myc-DDX5 constructs to ascertain level of expression. HeLa cells were co-transfected with different Myc-tagged DDX5 expressors (DDX5 wild type, DDX5-T176R + E178V + L179A and DDX5-E249Q). Cell lysates were harvested 48 h post transfection for Western blot using a Myc specific antibody. Mock or untransfected cell lysates were used as control. **c** Graphical abstract showing the proposed mechanistic action of DDX5 in HIV-1 transcription processivity

Given that DDX5 utilises distinct RNA binding motifs and the highly characterised Walker B motif is essential for DDX5′s role in potentiating HIV transcription, we performed co-immunoprecipitation experiments to determine whether the loss of function of DDX5-T176R + E178V + L179A and DDX5-E249Q was due to loss of interaction with HEXIM1. The respective Myc tagged constructs of DDX5 wild type (wt), DDX5-T176R + E178V + L179A and DDX5-E249Q were co-transfected with HIV-1 proviral plasmid (pLAI) in HeLa cells. We found that both DDX5-T176R + E178V + L179A and DDX5-E249Q lose the ability to co-immunoprecipitate with HEXIM1. This would be consistent with the interaction of DDX5 and HEXIM1 being RNA dependent (Fig. 8a). The different Myc tagged DDX5 constructs were well expressed as assessed by western blot (Fig. 8b).

## Discussion

HIV-1 transcription is a multistep process that is regulated and coordinated to promote efficient viral replication. Efficient Pol II processivity beyond the TAR region requires the presence of the Tat/P-TEFb complex [23, 46]. Tat/P-TEFb complex formation involves a series of dynamic transient interactions with different cellular factors, including snRNA. and sequential rearrangement of intermediate complexes.

Cellular P-TEFb is stored mainly in the inactive form sequestered with HEXIM1/2 bound to the 7SK snRNP complex, HIV-1 Tat competes with HEXIM1/2 to disrupt its binding on P-TEFb [23] however detailed understanding of the molecular mechanisms behind Tat dislodging HEXIM1/2 and then successfully preventing HEXIM1/2 from binding to free P-TEFb has been lacking. We show that DDX5 knockdown reduces HIV expression and that this can be rescued with exogenous DDX5. Overexpression of DDX5 significantly increases expression of HIV indicating that DDX5 is a powerful cofactor for Tat. Our co-immunoprecipitation data reveal that DDX5 can interact with both Tat and HEXIM1 within the cell and that it has a significant potentiating effect when co-expressed with a Tat expressing plasmid in TZM-bl cells (Fig. 3a–e) and in Hela cells (Additional file 3: Fig. S3a, b) providing a plausible scenario that DDX5/Tat interacts with the 7SK snRNP releasing P-TEFb and sequestering HEXIM to block its inhibitory effect. Consistent with this we could not detect DDX5 interacting with other P-TEFb components (Cyclin T1 or CDK9) (Additional file 2: Fig. S2a, b). We thus describe a novel component of this regulatory machine, DDX5, and add an additional layer to transcriptional control of HIV and show that this is specific for the Tat/LTR system and not a general enhancing effect on transcription by DDX5. Evidence of molecular mimicry between viral and host-protein-RNA complexes (Tat-TAR and HEXIM1-7SK snRNA) has been used to suggest that Tat competitively displaces HEXIM1 from 7SK snRNA [9]. The fact that DDX5 was recently identified by mass spectrometry as one of the LARP7 interactors reinforces our findings that DDX5 can interact with components of the 7SK snRNP complex and strengthens our evidence that it is critical for efficient HIV-1 replication.

DDX5 can exist as a monomer and a homodimer or can form heterodimers with its paralog DDX17. The two helicases have many functions in common. Based on the substitution findings (Fig. 4a, b) we conclude that the effects of DDX5 on HIV replication are independent of DDX17. Thus, an increase in DDX17 seen upon DDX5 depletion is not responsible for the phenotype produced by manipulating DDX5.

We delineated the DDX5 motifs and the biochemical activities that are essential for its role in HIV-1 replication. DDX5 utilises distinct RNA binding and ATPase motifs, namely: Q, 1a and 1b motifs (Fig. 5a–j) and the glycine doublet motif (Fig. 7a–d). The Walker A ATPase activity and the RNA binding activities of DDX5-S279L and DDX5-R431Q were dispensable for DDX5 mediated transactivation. Although the ATPase activity of the Walker A motif is dispensable, rather surprising is the requirement for the ATPase activity of the Q motif. The Q motif’s ability to efficiently bind ssRNA rather than its ATPase activity might be required [1, 41]. Therefore, although RNA and ATPase activities are essential these functions are mediated through specific RNA and ATP binding motifs by DDX5.

The Walker B (DEAD) motif (helicase motif) is indispensable yet, strikingly, the helicase activity of motif III is dispensable (Additional file 4: Fig. S4a, b). Mutations in motif III have been shown to cause minor effects on ATP binding, hydrolysis and RNA binding but cause a significant loss of helicase activity [47, 48].

Motif VI (HRIGRTGR), at the interface between domains 1 and domain 2, is important for ATPase activity and RNA binding. Mutating the arginine residue to glutamine abrogates both its ATPase and RNA binding activity [44, 48, 49] however the siDDX5A resistant-DDX5-R431Q mutant successfully restored HIV-1 infectivity indicating it is dispensable for HIV-1 replication (Additional file 5: Fig. S5a, c). Both DDX5-S279L and DDX5-R431Q in the siDDX5A rescue plasmid are successfully expressed (Additional file 5: Fig. S5d, e).

RNA binding motifs III and VI are independent of each other and independent of the RNA binding motifs 1a and 1b [1]. Therefore, our findings that both siDDX5A resistant mutants DDX5-S279L and DDX5-R431Q successfully restored HIV-1 infectivity suggest that neither motif III nor motif VI is required for DDX5 to affect HIV-1 replication. However, RNA binding motifs 1a and 1b are essential [1] and mutating one or the other resulted in failure to restore viral infectivity. This point towards DDX5′s role in HIV-1 transcription probably being independent of its transactivation domain (TAD) as both DDX5-S279L and DDX5-R431Q which are situated within the TAD are dispensable.

Identification of these functional motifs will be invaluable in further mapping and understanding the critical cofactors involved in the effect of DDX5 in facilitating and enhancing HIV expression. We have shown that RNA is required for interaction of DDX5 with HEXIM1 but whether this is cellular or viral RNA is as yet unclear. The mechanistic model we propose is consistent with DDX5 interacting with HIV-1 Tat and sequestering HEXIM1 thereby facilitating P-TEFb access to Tat to permit efficient HIV-1 transcription processivity (Fig. 8c).

## Conclusions

The DEAD box helicase DDX5 interacts with Tat/HEXIM1 and is necessary for HIV transcription. There is clear evidence that the role of DDX5 in HIV replication is independent of its paralog, DDX17. We show that the DDX5 motifs; Q, 1a, 1b, glycine doublet and Walker B are essential for DDX5′s role in HIV replication. The specific function of DDX5 to interact with HEXIM1 is essential for HIV-1 replication and may have more widespread implications in cell biology.

## Methods

### Cells and plasmids

HeLa M, a derivative of HeLa cells [50] were obtained from ATCC. TZM-bl cells, carrying two HIV-1 LTR-driven reporter genes, firefly luciferase and *E. coli* β-galactosidase [51, 52], is a HeLa cell clone that stably expresses high levels of CD4, CXCR4 and CCR5 receptors, was obtained from NIH AIDS Research and Reference Reagent Program. HeLa M and TZM-bl cells were grown in Dulbecco’s Modified Eagle Medium (DMEM) supplemented with 10% Fetal Bovine Serum (FBS). Jurkat cells (obtained from NIBSC Centre for AIDS Research London, UK) were grown in RPMI supplemented with 10% FBS. Generation of the siDDX5A resistant constructs was performed using the site directed mutagenesis (SDM) kit (Agilent). See Additional file 6: Table S1 for primer sequences. Myc tagged DDX5 and DDX17 were kind gifts from Dr Frances Fuller-Pace (Dundee). An empty cloning vector pBluescript (Stratagene) was used to maintain a constant amount of DNA in transfection experiments. pLAI is a full-length molecular clone of HIV-1 strain LAI for the expression of wild type virus [53]. pcDNA3.0-Tat is a Tat expressor under the CMV promoter (a kind gift of Dr J Sodroski, Harvard).

### Cell viability

CellTiter-Glo Luminescent Cell Viability Assay (Promega) was adapted for 96-well half-area plates and assay performed as previously described [54].

### siRNA and plasmid DNA transfections

siDDX3, siDDX5A, siDDX17 and siControl were purchased from Life Technologies. See Additional file 7: Table S2 for siRNA sequences. Sequential transfections were performed at 24 h (1 µl of 20 µM siRNA alone) and 48 h (siRNA + DNA plasmids) post seeding in 24 well plates. A constant amount, 200 ng of pLAI was used. In the rescue experiments, in addition to siDDX5A and pLAI, HeLa cells were co-transfected with increasing amounts of various siDDX5A resistant constructs. All transfections were carried out using jetPRIME (Polyplus) transfection reagent per the manufacturer’s protocol. Cells and supernatant were harvested 48 h post co-transfection and the supernatant was used to infect CD4 + TZM-bl cells to assay for viral infectivity. To assess siRNA mediated knockdown and rescue cell lysates were subjected to western blotting.

### Nucleofection

Amaxa^®^ Nucleofector^®^ II Device and Amaxa^®^ cell line Nucleofector^®^ Kit V (Cat. No. VCA 1003) from Lonza was used for nucleofection of Jurkat cells per the manufacturer’s protocol. Cells were sub-cultured in 12 well plates for 48 h and 1x10^6^ cells per condition were used for nucleofection with 2 µg DNA plasmid of either test sample or control plasmid (pmaxGFP^®^Vector) together with 30 pmol/sample siRNA (either siControl or siDDX5A). 48 h post nucleofection, medium was replaced with fresh RPMI 1640. Cell culture supernatant and cell lysates were harvested 96 h post nucleofection.

### Virus infectivity assay

Virus infectivity in culture supernatants was determined by infecting TZM-bl indicator cells, based on Tat-dependent upregulation of LTR-driven luciferase expression. Cell lysates were harvested 48 h post infection. 5 µl of cell lysate were transferred to a 96-well half-area plate, to which 25 µl of Luciferase Assay Reagent was added. Firefly luciferase activity was quantified using the Luciferase Assay System with the Glomax 96 Microplate Luminometer (Promega).

### Nuclear extracts preparation and co-immunoprecipitation

HeLa cells were co-transfected with the appropriate Myc-tagged constructs and pLAI. Cells were harvested 48 h post transfection and nuclear extracts prepared as previously described [55, 56] with the following buffers and modifications: Lysis buffer containing 10 mM HEPES pH 7.9; 1.5 mM MgCl_2_; 10 mM KCl; 0.5 mM DTT supplemented with protease inhibitors (Roche). This was followed by 5 µl per 500 µl reaction mix RNase (Sigma) treatment for 10 min at 4 °C. Samples were incubated overnight with 2 µg of either Myc or Isotype control antibody in CO-IP buffer (20 mM HEPES (pH 7.9); 150 mM NaCl; 0.5 mM DTT; 20% (v/v) Glycerol; 10 mM NaF and protease inhibitors). Samples were then incubated with A/G ultralink Sepharose beads (Thermoscientific) for 3 h at 4 °C. After washing with CO-IP buffer, samples were eluted in 2X Laemmli sample buffer. Proteins were separated by SDS-PAGE followed by western blotting using standard protocol.

### Western blotting

Proteins were separated by SDS-PAGE before being subjected to western blot. The following antibodies were from Santa Cruz: Myc (9E10), DDX17 (sc-398168), Abcam: HIV-Tat (ab43014), HEXIM1 (ab25388), Cyclin T1 (ab176702), CDK9 (ab6544), Rabbit Isotype matched control IgG (ab27478), Rabbit c-Myc (ab39688) and GAPDH (ab9485). DDX5 antibody PAb-204 was a kind gift from Dr Frances Fuller-Pace (Dundee). HIV-1 p55/p24 (ARP, NIBSC) [57]. Mouse IgG Isotype control (401402) was obtained from Biolegend. The following secondary antibodies were from: Cell Signalling: horseradish peroxidase (HRP)-conjugated anti-mouse (#7076, 1: 2000), Santa Cruz: HRP-conjugated anti-rabbit (#2123, 1: 2000). Detection was carried out using ECL prime (Amersham) per the manufacturer’s instructions.

### Enzyme-linked immunosorbent assay

Extracellular and intracellular/supernatant CA-p24 levels were quantified by ELISA (Alto) with slight modification [58].

### RT-qPCR

HeLa cells were sequentially transfected with siRNA and DNA plasmids as previously described in 24 well plates. Total RNA was extracted using RNeasy kit (Qiagen) per the manufacturer’s instructions. 5 µg of total RNA was used for complementary DNA synthesis with random hexamers, using reverse-transcription kit (Applied Biosystems). Applied Biosystems 7500 HT Fast Real Time PCR System (Life Technologies) and SYBR green qPCR reagent (Applied Biosystems) were used for qPCR. Primer sequences and thermo-cycling conditions have been previously described [59, 60] and are shown in Additional file 6: Table S1 and Additional file 8: Table S3 respectively. The specificity of qPCR products was examined by a dissociation curve and relative abundance of transcripts was calculated using the 2^−∆∆CT^ method [61]. Results were normalised to Actin and mean results of triplicates were converted to ratios relative to siControl samples.

### Statistical analyses

Statistical analyses were performed in Excel and GraphPad Prism version 4. The data for viral infectivity and CA-p24 production were analysed by unpaired two-tailed Student’s *t* test, with Welch’s correction.

## Supplementary information

**Additional file 1. Figure S1.** Cell viability. (A and B) Cells were seeded in a 96 well at 4 × 10^3^ cells per well and sequentially transfected with 10 pmol of siControl or siDDX5A and then a second round of siRNA together with or without increasing concentrations of siDDX5A rescue constructs. The number of viable cells was determined by measuring the amount of ATP present as an indicator of metabolically active cells. This was then calculated as a percentage relative to untransfected cells. Puromycin treated cells were used as positive controls. Bars represent mean of duplicate samples ± SEM.

**Additional file 2: Figure S2.** DDX5 does not co-immunoprecipitate with P-TEFb (Cyclin T1 and CDK9). (A and B) HeLa cells were co-transfected with Myc-DDX5 and pLAI. Cell lysates were harvested 48 h post co-transfection for co-immunoprecipitation. A Myc specific antibody or isotype matched control antibody (IgG) was used for pull-down. Samples were then subjected to SDS-PAGE and western blotting by probing for Cyclin T1 and CDK9 respectively. Positive control for Myc-DDX5 pulldown was confirmed by the pulldown of Myc-DDX5 and immunoblotting for DDX17 (Fig. S4) as the two are known to form heterodimers in cells.

**Additional file 3: Figure S3.** DDX5 specifically potentiates Tat driven LTR luciferase activity. (A) HeLa cells were co-transfected with constant amount of 3′LTR-Luciferase and Tat expressors plus increasing concentration of DDX5. Cell lysates were harvested 48 h and luciferase activity measured using the luciferase assay. The graph is a representative of three independent experiments done in triplicate. Bars represent mean of triplicate samples ± SEM. (B) Western blot showing the overexpression of DDX5. (C) Hela cells were transfected with constant amount of 3′LTR-Luciferase and Tat expressors plus either siControl or siDDX5A. Cell lysates were harvested after 72 h and luciferase activity measured using the luciferase assay. The graph is a representative of three independent experiments done in triplicate. Bars represent mean of triplicate samples ± SEM. Statistical significance *P < 0.05. (D) Hela cells were co-transfected with a constant amount of pEF-Luciferase plus increasing concentration of DDX5 expressor. Cell lysates were harvested after 72 h and luciferase activity measured using the luciferase assay. (E) Hela cells were co-transfected with a constant amount of pcDNA3.1-Luciferase plus increasing concentration of DDX5 expressor. Cell lysates were harvested after 72 h and luciferase activity measured using the luciferase assay. (F) Hela cells were co-transfected with a constant amount of pJL-Luciferase plus increasing concentration of DDX5 expressor. Cell lysates were harvested after 72 h and luciferase activity measured using the luciferase assay. (G) Western blot showing the overexpression of DDX5 and GAPDH as loading control in cell lysates from (D). (H) Western blot showing the overexpression of DDX5 and GAPDH as loading control in cell lysates from (E). (I) Western blot showing the overexpression of DDX5 and GAPDH as loading control in cell lysates from (F). The graphs, (D, E and F) are from two independent experiments done in triplicates for each different luciferase expressor. Bars represent mean of triplicate samples ± SEM. Statistical significance ****P < 0.0001.

**Additional file 4: Figure S4.** DDX5 interacts with DDX17 and effects of DDX5 overexpression on endogenous DDX17 levels. (A) HeLa cells were co-transfected with Myc-DDX17. Cell lysates were harvested 48 h post co-transfection for co-immunoprecipitation. A Myc specific antibody or isotype matched control antibody (IgG) was used for pull-down. Samples were subjected to SDS-PAGE and western blotting by probing for DDX5. (B) HeLa cells were co-transfected with Myc-DDX5. Cell lysates were harvested 48 h post co-transfection for co-immunoprecipitation. A Myc specific antibody or isotype matched control antibody (IgG) was used for pull-down. Samples were subjected to SDS-PAGE and western blotting by probing for DDX17. (C) Effect of overexpressing DDX5 wild type on the expression level of endogenous DDX17. HeLa cells were co-transfected with a constant amount of pLAI and increasing concentrations of DDX5 wild type expressor construct. Cell lysates were harvested 48 h post co-transfection followed by western blotting for DDX5 and DDX17.

**Additional file 5: Figure S5.** DDX5 ATPase and RNA binding motifs mutants are essential for Tat activity. (A) Schematic representation of DDX5 and the annotated individual point mutations that render DDX5 defective for RNA binding activities in motifs III and IV. (B) Effect on HIV-1 infectivity following endogenous DDX5 depletion and rescue with DDX5-S279L. Cells were sequentially transfected with siDDX5A and then a second round of siRNA together with the replication competent HIV-1 proviral clone pLAI, with or without increasing concentrations of siDDX5A resistant DDX5-S279L expressor construct. Supernatant was harvested 48 h post co-transfection and used to infect CD4 + TZM-bl indicator cells for assessing viral infectivity. TZM-bl cell lysates were harvested 48 h post infection and luciferase activity quantified. (C) Effect on HIV-1 infectivity following endogenous DDX5 depletion and rescue with DDX5-R431Q. Cells were sequentially transfected with siDDX5A and then a second round of siRNA together with the replication competent HIV-1 proviral clone pLAI, with or without increasing concentrations of siDDX5A resistant DDX5-R431Q expressor construct. Supernatant was harvested 48 h post co-transfection and used to infect CD4 + TZM-bl indicator cells for assessing viral infectivity. TZM-bl cell lysates were harvested 48 h post infection and luciferase activity quantified. Each graph is a representative of at least two independent experiments done in triplicate. Bars represent mean of triplicate samples ± SEM. (D) Western blot showing expression of DDX5 following knockdown and rescue with DDX5-S279L. (E) Western blot showing DDX5 expression following knockdown and rescue with DDX5-R431Q. Statistical significance *P < 0.05, **P < 0.01.

**Additional file 6: Table S1.** List of siRNAs.

**Additional file 7: Table S2.** List of PCR and RT-qPCR.

**Additional file 8: Table S3.** RT-qPCR thermocycling conditions.

## Data Availability

All data generated or analysed during this study are included in this published article [and its additional files].

## Abbreviations

HIV: Human immunodeficiency virus
P-TEFb: Positive transcription elongation factor b
HEXIM1/2: Hexamethylene bisacetamide-inducible proteins 1/2
siRNA: Small interfering RNA
qPCR: Quantitative polymerase chain reaction
CDK9: Cyclin-dependent kinase 9
