# Supplementary material for: DDX5 potentiates HIV-1 transcription as a co-factor of Tat
Source: Retrovirology. 2020 Mar 30;17:6. doi: 10.1186/s12977-020-00514-4 (PMC7106839; doi:10.1186/s12977-020-00514-4)
Supplement: Supplementary file 6 — Additional file 6: Table S1. List of siRNAs. [file 12977_2020_514_MOESM6_ESM.docx]

**Table S1. List of siRNAs**

| Helicase | Sense | Antisense |
| --- | --- | --- |
| DDX5 | UUUAGAACUGGCUUCGGGCAGUUGU | ACAACUGCCCGAAGCCAGUUCUAAA |
| DDX17 | CGUAUCUCCUGCCUGCAAUTT | AUUGCAGGCAGGAGAUACGTT |
| DDX3 | GAUUCACUGACCUUAGUGUTT | ACACUAAGGUCAGUGAAUCTT |
