# Supplementary material for: DDX5 potentiates HIV-1 transcription as a co-factor of Tat
Source: Retrovirology. 2020 Mar 30;17:6. doi: 10.1186/s12977-020-00514-4 (PMC7106839; doi:10.1186/s12977-020-00514-4)
Supplement: Supplementary file 7 — Additional file 7: Table S2. List of PCR and RT-qPCR. [file 12977_2020_514_MOESM7_ESM.docx]

**Table S2. List of PCR and RT-qPCR**

| Primer name | Primer sequence (5’ to 3’) |
| --- | --- |
| DDX5-K144A Fwd | GGCACAGACTGGATCTGGGGCAACATTGTCTTATTTGCTTC |
| DDX5-K144A Rev | GAAGCAAATAAGACAATGTTGCCCCAGATCCAGTCTGTGCC |
| DDX5-E249Q Fwd | CCTACCTTGTCCTTGATCAAGCAGATAGAATGCTTG |
| DDX5-E249Q Rev | CAAGCATTCTATCTGCTTGATCAAGGACAAGGTAGG |
| DDX5-Q121A Fwd | CACTGAACCCACTGCTATTGCAGCTCAGGGATGGCCAGTTGC |
| DDX5-Q121A Rev | GCAACTGGCCATCCCTGAGCTGCAATAGCAGTGGGTTCAGTG |
| DDX5-T176R, E178V,L179A–(Motif 1a) Fwd | GGTGCTGGCACCAAGGCGGGTAGCGGCCCAACAGGTGCAGC |
| DDX5-T176R, E178V,L179A–(Motif 1a) Rev | GCTGCACCTGTTGGGCCGCTACCCGCCTTGGTGCCAGCACC |
| DDX5-G203D Fwd | GTCTACTTGTATCTACGATGGTGCTCCTAAGGGACC |
| DDX5-G203D Rev | GGTCCCTTAGGAGCACCATCGTAGATACAAGTAGAC |
| DDX5-T224D Fwd | GGAAATCTGTATTGCAGATCCTGGAAGACTGATTG |
| DDX5-T224D Rev | CAATCAGTCTTCCAGGATCTGCAATACAGATTTCC |
| DDX5-S279L Fwd | GCAAACTCTAATGTGGCTCGCGACTTGGCCAAAAG |
| DDX5-S279L Rev | CTTTTGGCCAAGTCGCGAGCCACATTAGAGTTTGC |
| DDX5-R431Q Fwd | CATCGAATTGGACAAACTGCTCGCAGTACC |
| DDX5-R431Q Rev | GGTACTGCGAGCAGTTTGTCCAATTCGATG |
| Unspliced RNA Fwd | GAC GCT CTC GCA CCC ATC TC |
| Unspliced RNA Rev | CTG AAG CGC GCA CGG CAA |
| Partially-spliced RNA Fwd | GGC GGC GAC TGG AAG AAG C |
| Partially-spliced RNA Rev | CTA TGA TTA CTA TGG ACC ACA C |
| Fully-spliced RNA Fwd | GAC TCA TCA AGT TTC TCT ATC AAA |
| Fully-spliced RNA Rev | AGT CTC TCA AGC GGT GGT |
| Beta Actin Fwd | GAGCGGTTCCGCTGCCCTGAGGCACTC |
| Beta Actin Rev | GGGCAGTGATCTCCTTCTGCATCCTG |
