# Supplementary material for: DDX5 potentiates HIV-1 transcription as a co-factor of Tat
Source: Retrovirology. 2020 Mar 30;17:6. doi: 10.1186/s12977-020-00514-4 (PMC7106839; doi:10.1186/s12977-020-00514-4)
Supplement: Supplementary file 8 — Additional file 8: Table S3. RT-qPCR thermocycling conditions. [file 12977_2020_514_MOESM8_ESM.docx]

**Table S3. RT-qPCR thermocycling conditions**

| Step 1 | 95 ºC for 5 min |
| --- | --- |
| Step 2 | 95 ºC for 15 sec |
| Step 3 | 60 ºC for 15 sec |
| Step 4 | 72ºC for 15 sec |
| Repeat Steps 2-4 | X40 |
| Step 5 | Melting curve to verify |
